# Synergistic Nitrogen Removal and Community Interaction Mechanism of Immobilized Bacteria Algae Symbiosis System

**DOI:** 10.3390/molecules31101764

**Published:** 2026-05-21

**Authors:** Jianyang Song, Peng Xu, Zhiheng Wei, Huimin Yao, Aohan Wang, Changfeng Xu, Yawei Zhu, Rongrong Wang, Xinfang Yuan

**Affiliations:** 1 Nanyang Key Laboratory of Water Pollution Control and Solid Waste Resource, Nanyang Institute of Technology, Nanyang 473004, China; 17870357018@163.com (P.X.); 15503836786@163.com (Z.W.); m73426980@163.com (H.Y.); 17629890636@163.com (A.W.); 15238541174@163.com (C.X.); zhuyw1990@126.com (Y.Z.); wrrong77@163.com (R.W.); seven20050911@163.com (X.Y.); 2 School of Civil Engineering, Nanyang Institute of Technology, Nanyang 473004, China; 3 School of Materials and Chemical Engineering, Zhengzhou University of Light Industry, Zhengzhou 450001, China; 4 School of Biological and Chemical Engineering, Nanyang Institute of Technology, Nanyang 473004, China

**Keywords:** aerobic denitrification, immobilization, biological denitrification, algal bacterial symbiotic system, denitrification efficiency

## Abstract

Ammonium nitrogen pollution presents a significant challenge in wastewater treatment. Traditional activated sludge processes often suffer from limitations such as low efficiency and high energy consumption when treating high-ammonium nitrogen wastewater. This study utilized previously screened high-efficiency heterotrophic nitrification aerobic denitrification (HN-AD) bacterial strains (*Pseudomonas alcaliphila* and *Paracoccus versutus*) synergistically with microalgae to construct an immobilized bacteria algae symbiotic system (IBAS). The nitrogen removal performance and microbial community response of the system were investigated under different nitrogen sources, carbon to nitrogen (C/N) ratios, and light intensities. Results demonstrated that the system achieved a removal rate of over 95% for nitrite and nitrate. Under conditions of C/N = 15 and high light intensity (335.36 μmol/(m^2^ · s)), the removal rates of NH_4_^+^-N, TN, and COD exceeded 90% without nitrite accumulation. Microbial community analysis revealed that high C/N conditions significantly enriched HN-AD functional bacteria (such as *Acinetobacter*) in the *Pseudomonadota* phylum and *Gammaproteobacteria* class. High light intensity promoted the proliferation of microalgae (*Chlorella* and *Halochlorella*), enhanced algal bacterial interaction, and improved system stability. This study elucidated the nitrogen removal mechanism of the IBAS under multi-factor regulation, providing a theoretical foundation and demonstrating application potential for low-carbon and high-efficiency wastewater treatment technologies.

## 1. Introduction

Ammonium nitrogen pollution remains a persistent and severe challenge in the field of wastewater treatment [1]. With the rapid increase in industrial and agricultural wastewater discharge, traditional activated sludge processes increasingly reveal shortcomings such as high energy consumption, substantial sludge production, and weak resistance to shock loads when treating high-ammonium nitrogen wastewater [2,3], particularly exhibiting limited nitrogen removal efficiency for wastewater with low carbon-to-nitrogen (C/N) ratios. Heterotrophic nitrification–aerobic denitrification (HN-AD) bacteria possess periplasmic nitrate reductase (NAP), which is insensitive to oxygen [4]. This capability enabled simultaneous nitrification and denitrification in aerobic environments [5,6,7]. Consequently, they exhibit enhanced adaptability for nitrogen removal across diverse conditions [8]. Previous studies have reported that co-cultivation of microalgae with activated sludge improves nutrient removal and promotes biofilm formation [9]. Microalgae consumed CO_2_ and produced oxygen through photosynthesis [10], while also influencing wastewater pH to some extent [11,12]. Recent advances in understanding these mutualistic interactions have further highlighted the potential of algal bacterial systems for sustainable wastewater treatment [13]. Compared to monocultures of microalgae or bacteria, microalgae bacteria symbiotic systems exhibit enhanced nutrient removal, biomass production, and microbial activity.

However, there are inherent limitations in relying solely on naturally occurring algae bacteria associations. These include uncontrollable abundances of functional bacteria and poor environmental adaptability, which often render such systems unstable under dynamic water quality conditions and restrict their application. Immobilization technology is widely recognized as a potential solution to address issues such as low survival rates and functional instability of free HN-AD bacteria in complex wastewaters. Jin, et al. [14] utilized hydrogel carriers loaded with microorganisms to enhance nitrogen removal performance, but the insufficient mechanical strength and mass transfer limitations of gel carriers hindered engineering applications. Porous ceramsite, with its stable mechanical strength and porous structure, serves as a promising alternative material [15]. Integrating immobilization technology to foster a deep combination of ceramsite with HN-AD bacteria, while introducing microalgae, holds significant promise for wastewater treatment. Nitrogen is an essential nutrient for microalgal growth. The nitrogen uptake mechanisms of microalgae are closely related to the nitrogen source, leading to a preference for certain nitrogen forms. Typically, ammonium (NH_4_^+^) was the primary form, and its assimilation requires the least energy consumption compared to other nitrogen sources like NO_3_^−^ and NO_2_^−^ [16]. The carbon-to-nitrogen ratio played a crucial role in the IBAS. Microalgae possess the ability to remediate and absorb pollutants such as carbon, nitrogen, and phosphorus from wastewater [17], while bacteria consume carbon sources during denitrification. Thus, an appropriate C/N ratio is critical for optimizing system functionality. Wang, et al. [18] showed that light intensity significantly impacts photosynthesis. External light sources can effectively promote microalgal function. The IBAS has garnered attention as a green and low-carbon alternative technology. It establishes a mutualistic relationship between microalgae and bacteria through an efficient resource-recycling loop. The microalgae assimilated metabolic CO_2_ and contaminants (N, P) from the bacteria. In return, the oxygen from algal photosynthesis supported bacterial aerobic respiration as a terminal electron acceptor. This synergistic interaction ultimately promotes wastewater treatment [19,20]. Nevertheless, the pollutant removal efficiency and key functional microorganisms involved in immobilized bacteria algae symbiotic systems require further investigation.

Relying on two efficient nitrogen-removing bacterial strains (*Pseudomonas alcaliphila* and *Paracoccus versutus*) screened and characterized in our previous study [21], an immobilized bacteria algae symbiotic system was established in the present work, which aimed to provide a novel solution for the challenge of stable nitrogen removal. The objectives of this study were as follows: (1) explore the removal effect of the system on different nitrogen sources; (2) evaluate the effect of light intensity and C/N ratio on pollutant removal; and (3) identify the key functional microorganisms involved in the denitrification process of the bacteria algae symbiosis system.

## 2. Results and Discussion

### 2.1. Exploration of Algal Growth Changes

**Figure 1:**
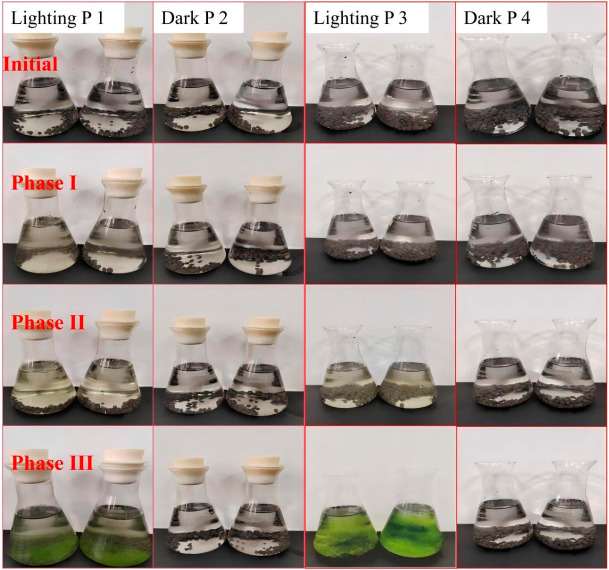
The typical forms of algae bacterial system under different conditions within a 30-day experimental period of phase I (1–10 days), phase II (11–20 days) and phase III (21–30 days).

Microalgal photosynthetic growth is the core driving force for the synergistic nitrogen removal of the immobilized bacteria algae symbiotic system (IBAS). Its biomass accumulation, growth cycle and photosynthetic activity directly determine the oxygen supply efficiency, carbon oxygen cycle intensity, and synergistic coupling effect with heterotrophic nitrification aerobic denitrification (HN-AD) bacteria in the system. Existing studies have shown that light conditions are the primary environmental factor regulating the photoautotrophic growth of microalgae, while the gas exchange state (sealed/open) of the culture system further regulates the growth rate and biomass peak of microalgae by affecting CO_2_ supply, metabolic waste emission and microenvironment stability. The typical forms of the algae bacterial system under different conditions over the 30-day experimental period are presented in Figure 1.

In the initial stage, all groups were clear and transparent, indicating that the biomass of the algae was extremely low and no obvious growth had begun. In Phase I (1–10 days, growth initiation period), in the first 10 days of culture, the light groups (P1, P3) and the dark groups (P2, P4) showed extremely significant growth phenotypic differentiation. The culture systems of groups P1 and P3 began to show slight turbidity, and the color gradually changed from the initial colorless and transparent to pale yellow-green to light green. The chromaticity change in the open-cultured P3 group was slightly earlier than that of the sealed-cultured P1 group. Quantitative detection of chlorophyll-a showed that the chlorophyll-a concentration of group P3 increased from the initial 0.5 mg/L to 8.7 ± 0.4 mg/L, and that of group P1 increased to 6.2 ± 0.3 mg/L. Both groups achieved significant accumulation of algal cell biomass, indicating the successful initiation of microalgal photoautotrophic growth. In sharp contrast, the culture systems of the P2 and P4 groups treated with full darkness remained clear and transparent throughout the period, the chlorophyll-a concentration was always maintained at the initial level without significant increase, and the algal cells did not proliferate effectively. These results fully confirm that light is a necessary prerequisite for microalgae to initiate photoautotrophic growth. As photosynthetic autotrophic microorganisms, the growth and reproduction of microalgae completely depend on photosynthesis driven by light reaction [22,23]. They capture light energy through photosystem II, fix CO_2_ into organic carbon, and complete cell material synthesis and proliferation [24]. In dark conditions, the light reaction of microalgae is completely inhibited, and carbon fixation and energy supply cannot be achieved through the Calvin cycle. Even if there is a small amount of organic carbon source in the system, it is difficult to support its long-term autotrophic growth, so effective biomass accumulation cannot be achieved, which is highly consistent with the research conclusion of Schultze, et al. [25]. At the same time, the growth performance of group P3 was better than that of group P1 at this stage, which preliminarily indicated that the gas exchange conditions of the open system were more conducive to the growth initiation of microalgae, which was directly related to the supply efficiency of CO_2_: the carbon fixation process of microalgae photosynthesis requires continuous CO_2_ supply, and the open system can directly exchange gas with the atmosphere to continuously supplement the CO_2_ consumed in the system, while the CO_2_ reserve in the sealed system is limited and is rapidly consumed with the progress of photosynthesis, which then becomes a limiting factor for microalgae growth [12,26].

In Phase III (21–30 days, stable growth period), from day 21 to day 30 of culture, the growth of microalgae in the light group gradually entered the stable platform period, the biomass accumulation tended to be flat, and the phenotypic characteristics were further differentiated. The culture system of group P3 turned into uniform dark green, the algal cell density reached the peak, the chlorophyll-a concentration was stable at 34.2 ± 0.7 mg/L, the system had good uniformity, and no obvious algal sedimentation or agglomeration occurred; the system of group P1 showed a rich green color, a small amount of algal cell aggregates appeared locally, and the chlorophyll-a concentration was stable at 24.5 ± 0.5 mg/L, with a biomass peak was significantly lower than that of group P3. The P2 and P4 groups treated with full darkness remained clear and transparent throughout the experimental period, with no significant difference from the initial stage, and the chlorophyll-a concentration was continuously maintained at an extremely low level, which completely failed to achieve the autotrophic growth and biomass accumulation of algal cells. The results of this stage showed that the open system can not only improve the proliferation rate of microalgae, but also optimize the stability of its long-term growth and extend the duration of the stable growth period. Group P3 could still maintain stable biomass and photosynthetic activity in this stage without obvious cell decay, which benefited from the excellent microenvironment regulation ability of the open system: continuous gas exchange ensured the stable supply of CO_2_ and timely discharge of metabolic waste, and the open system was more conducive to the uniform distribution of light, avoiding the light shielding effect under high algal cell density, so that most algal cells could obtain sufficient light and maintain efficient photosynthetic activity [20,27,28,29]. In the sealed cultured group P1, local algal aggregation occurred. On the one hand, the lack of nutrients and CO_2_ in the system led to the algal cells entering the decline phase, and the secreted extracellular polymers caused cell aggregation and sedimentation; on the other hand, the microenvironment imbalance in the sealed system, the drastic fluctuation of pH and dissolved oxygen further aggravated the physiological stress of algal cells, leading to the decline of their growth stability [30,31].

Group P3 could still maintain stable biomass and photosynthetic activity in this stage without obvious cell decay, which benefited from the excellent microenvironment regulation ability of the open system: continuous gas exchange ensured the stable supply of CO_2_ and timely discharge of metabolic waste [32,33,34], and the open system was more conducive to the uniform distribution of light, avoiding the light shielding effect under high algal cell density, so that most algal cells could obtain sufficient light and maintain efficient photosynthetic activity. In the sealed cultured group P1, local algal aggregation occurred. On the one hand, the lack of nutrients and CO_2_ in the system led to the algal cells entering the decline phase, and the secreted extracellular polymers caused cell aggregation and sedimentation; on the other hand, the microenvironment imbalance in the sealed system, the drastic fluctuation of pH and dissolved oxygen further aggravated the physiological stress of algal cells, leading to the decline of their growth stability [35,36].

In summary, light was the key driver of autotrophic algal growth. Over time, only the illuminated group exhibited a process of “growth initiation → accelerated proliferation → massive accumulation”, while the dark-treated group, with inhibited photosynthesis, failed to grow effectively. Minor differences in apparatus (e.g., sealing methods, carrier morphology) exerted only secondary effects on the growth rate and biomass accumulation of the illuminated group.

### 2.2. Study on Decontamination Effect of IBAS on Different Concentrations and Morphologies of Nitrogen Sources

The synergistic response rules of the IBAS to different nitrogen morphologies and concentration gradients, as well as the interaction between bacterial denitrification and algal assimilation under different substrate conditions, have not been systematically clarified. To fill this gap, batch experiments were conducted with three typical inorganic nitrogen sources (NH_4_^+^-N, NO_3_^−^-N, NO_2_^−^-N) as the sole nitrogen substrate, each set with three concentration gradients (50, 70, 100 mg/L) covering the common concentration range of actual high-ammonia wastewater. The nitrogen removal dynamics, total nitrogen (TN) removal efficiency, and chemical oxygen demand (COD) consumption characteristics of IBAS within 48 h were systematically investigated, to clarify the substrate preference, anti-shock load capacity, and underlying metabolic mechanism of the system.

**Figure 2:**
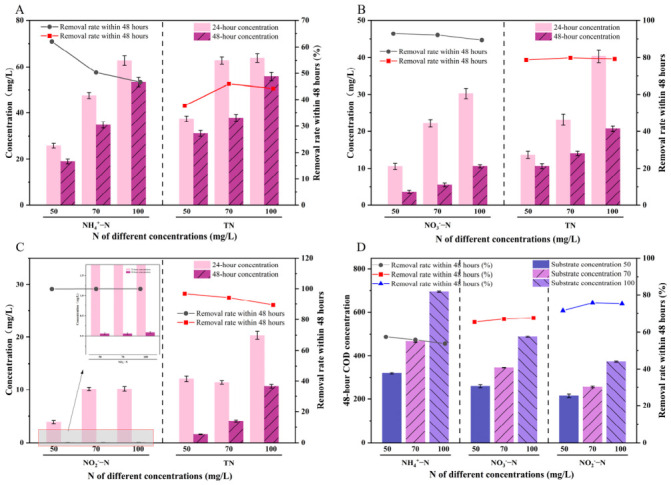
IBAS denitrification effect of different substrates: (**A**) Substrate NH_4_^+^-N; (**B**) Substrate NO_3_^−^-N; (**C**) Substrate NO_2_^−^-N; (**D**) COD removal effect.

When NH_4_^+^-N was used as the sole nitrogen source, the nitrogen removal efficiency of IBAS showed a significant concentration-dependent inhibitory effect, as presented in Figure 2A. At the initial NH_4_^+^-N concentration of 50 mg/L, the system achieved a 70% NH_4_^+^-N removal rate within 48 h, with a corresponding TN removal rate of 68.2%, indicating that most of the removed ammonium nitrogen was converted to gaseous nitrogen through the HN-AD pathway, rather than just being assimilated by microalgae and bacteria for biomass synthesis. When the initial concentration increased to 70 mg/L, the NH_4_^+^-N and TN removal rates slightly increased to 72.4% and 70.1%, respectively, suggesting that the substrate concentration within this range could meet the metabolic demand of functional microorganisms without causing significant inhibition. However, when the initial NH_4_^+^-N concentration further rose to 100 mg/L, the NH_4_^+^-N removal rate dropped sharply to approximately 50%, and the TN removal rate also decreased significantly to 46.8%, indicating that the system suffered from severe substrate inhibition under high ammonium loading.

This inhibitory effect was mainly attributed to the toxicity of free ammonia (FA) to functional microorganisms. Under the experimental pH condition (7.5 ± 0.2), the FA concentration in the 100 mg/L NH_4_^+^-N system exceeded 5 mg/L, which surpassed the inhibition threshold for most HN-AD bacteria [37,38,39]. High concentration of FA can inactivate the key enzymes of heterotrophic nitrification, including ammonia monooxygenase (AMO) and hydroxylamine oxidase (HAO) [37], which directly blocks the conversion of NH_4_^+^-N to hydroxylamine and subsequent nitrite/nitrate, thus reducing the nitrification efficiency of the system. Meanwhile, high ammonium stress also inhibited the photosynthetic activity of microalgae, reduced the oxygen supply for bacterial aerobic denitrification, and broke the synergistic carbon oxygen cycle between algae and bacteria, further aggravating the deterioration of nitrogen removal performance [40,41,42]. Consistent with the nitrogen removal results, the COD consumption of the system increased continuously with the increase in NH_4_^+^-N concentration (Figure 2D). At 100 mg/L NH_4_^+^-N, the COD consumption rate reached 82.3%, which was 21.7 percentage points higher than that at 50 mg/L. This phenomenon indicated that under high ammonium stress, microorganisms consumed a large amount of organic carbon for stress resistance and endogenous metabolism to maintain cell activity, rather than using it as an electron donor for denitrification, resulting in the decoupling of carbon consumption and nitrogen removal.

When NO_3_^−^-N was used as the sole nitrogen source, the nitrogen removal curve of IBAS showed a unique valley-shaped trend with the increase in initial concentration (Figure 2B). At the initial concentration of 50 mg/L, the 48 h NO_3_^−^-N removal rate reached 83.6%, with a TN removal rate of 81.2%; when the concentration increased to 70 mg/L, the NO_3_^−^-N and TN removal rates decreased to the lowest point of 72.1% and 70.5%, respectively; while when the concentration further rose to 100 mg/L, the removal efficiency rebounded significantly, with NO_3_^−^-N and TN removal rates recovering to 85.4% and 82.7%, respectively, accompanied by a significant increase in COD consumption (Figure 2D).

This special valley-shaped concentration response was mainly determined by the dual effects of substrate induction and substrate limitation of nitrate metabolic enzymes. At low concentration (50 mg/L), the nitrate substrate could meet the basic metabolic demand of HN-AD bacteria, and the periplasmic nitrate reductase (NAP) responsible for aerobic denitrification maintained a stable expression level, so the system maintained a good nitrate removal performance [43,44]. At 70 mg/L, the substrate concentration was not sufficient to induce high expression of NAP, but exceeded the optimal adaptation range of the constitutively expressed enzyme, resulting in a decrease in overall reduction efficiency [39,40]. When the concentration reached 100 mg/L, high concentration of nitrate significantly induced the up-regulated expression of NAP and nitrite reductase (NIR) in HN-AD bacteria, enhanced the electron transport activity of the denitrification pathway, and thus improved the nitrate reduction efficiency. In addition, compared with the NH_4_^+^-N system, the NO_3_^−^-N system had no obvious high-concentration toxicity inhibition, which was because nitrate nitrogen would not produce FA-like toxic intermediates, and had a wider adaptation concentration range for functional microorganism [38]. It should be noted that the overall nitrate removal efficiency of the system was lower than that of nitrite, which was due to the more complex metabolic steps of nitrate denitrification: nitrate needs to be reduced to nitrite by NAP first, and then further reduced to gaseous nitrogen by NIR, which requires more electron donors and has a longer reaction path, resulting in a relatively slower removal rate [45].

The outstanding nitrite removal performance of IBAS mainly came from the metabolic advantage of the short-cut denitrification pathway. Different from the complete denitrification pathway of nitrate, nitrite can be directly reduced to gaseous nitrogen by NIR in HN-AD bacteria, skipping the rate-limiting step of nitrate reduction. This not only shortens the metabolic path and reduces the energy loss in the electron transfer process, but also avoids the accumulation of intermediate products, thus achieving higher electron utilization efficiency and faster nitrogen removal rate. In addition, the immobilized carrier provided a stable microenvironment for functional microorganisms: the porous ceramsite formed a natural aerobic-anoxic gradient from the surface to the interior, which not only protected the oxygen-sensitive NIR enzyme from inactivation, but also provided a suitable niche for aerobic denitrifying bacteria and microalgae to grow synergistically, further enhancing the stability of nitrite removal. Meanwhile, the NH_4_^+^-N pathway posed both challenges and opportunities for process intensification. This made it a prominent focus in the subsequent research on reaction scale. The obvious sensitivity of the ammonium pathway to concentration stress highlighted the necessity of optimizing operating conditions (such as dissolved oxygen control, pH regulation and sludge retention time) to alleviate inhibition and promote the full coupling of nitrification and denitrification. Therefore, focusing subsequent reactor research on NH_4_^+^-N removal holds practical significance for treating real-world wastewater, which is typically ammonium-dominant. Moreover, it provided a realistic basis for understanding how reactors incorporating bio-enhanced ceramic media mitigate ammonium inhibition, enhance process stability, and maximize TN removal under continuous flow conditions. This targeted approach bridged the gap between the promising dynamics of batch operations and the scalable and effective nitrogen management.

### 2.3. Analysis of Decontamination Effect of IBAS Under Different Influencing Factors

**Figure 3:**
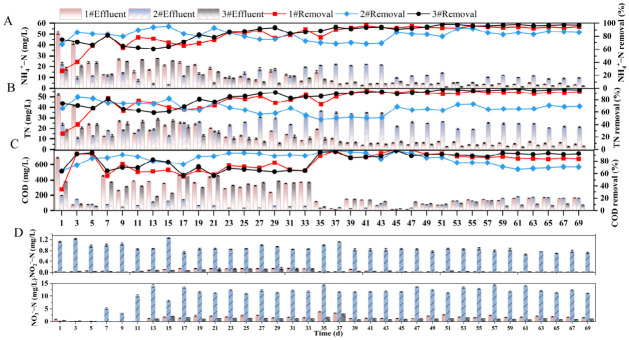
Variation in pollutant removal in reaction system 1#, 2# and 3# with operating time: (**A**) COD removal; (**B**) NH_4_^+^-N removal; (**C**) TN removal; (**D**) NO_3_^−^-N and NO_2_^−^-N changes.

The performance of IBAS was systematically evaluated under different C/N and light conditions. To clarify the effects of the C/N ratio and light intensity on pollutant removal during the treatment of high ammonium nitrogen wastewater by IBAS, the temporal dynamics and removal efficiencies of pollutants (e.g., NH_4_^+^-N, NO_3_^−^-N) were illustrated in Figure 3.

As shown in Figure 3, system 3# (C/N = 15, high light intensity) achieved the optimal and stable removal of NH_4_^+^-N (95%), TN (95%), and COD (90%), with no accumulation of NO_3_^−^-N or NO_2_^−^-N. This superior performance is attributed to light-enhanced symbiotic interactions. High light intensity promoted the photosynthesis of microalgae, providing sufficient oxygen for immobilized HN-AD bacteria to achieve efficient aerobic nitrification and denitrification [46]. Simultaneously, sufficient organic carbon supported bacterial metabolism, while CO_2_ released from bacterial respiration was rapidly assimilated by microalgae. This carbon oxygen exchange not only reduced greenhouse gas emissions but also synchronized nitrification and denitrification rates. This effectively eliminated the accumulation of intermediate products and maximized nitrogen removal [47].

In contrast, 2# (C/N = 8, low light intensity) exhibited unstable and incomplete removal of all pollutants, with significant accumulation of NO_3_^−^-N and intermittent peaks of NO_2_^−^-N. The lack of carbon source limited the electron supply for the denitrification process, while low light intensity simultaneously reduced algal photosynthesis, resulting in decreased oxygen production and the disruption of bacteria algae synergy. The breakdown of the O_2_-CO_2_ exchange cycle led to the imbalance between nitrification and denitrification, as well as low TN removal efficiency.

In reactor 1#, the removal of NH_4_^+^-N showed relatively stable performance, and the NH_4_^+^-N removal efficiency of the IBAS could reach over 90% during its stable operation period. During this process, HN-AD bacteria degraded NH_4_^+^-N, while microalgae, limited by light intensity, provided limited O_2_ for the aerobic denitrification of bacteria. In reactor 3# (with the same C/N ratio as reactor 1# but higher light intensity), the photosynthesis of microalgae was enhanced, providing abundant O_2_ for HN-AD bacteria and accelerating ammonium oxidation/nitrification. Therefore, in the later stage, the NH_4_^+^-N removal efficiency of reactor 3# was superior to that of reactor 1#, demonstrating the high light-driven synergistic effect between algal O_2_ production and bacterial NH_4_^+^-N degradation. For reactor 2# (low C/N + low light intensity), the insufficient carbon source limited electron supply for bacterial denitrification and microalgal growth (due to limited inorganic carbon produced by bacterial respiration), resulting in fluctuating and low NH_4_^+^-N removal efficiency. Although sufficient carbon was adequate to support the partial nitrification-denitrification of bacteria, the limited light intensity restricted the activity of microalgae and the production of oxygen. This insufficient light led to a small accumulation of nitrogen intermediates and unsatisfactory COD utilization efficiency, indicating that even with sufficient organic carbon, light remained an indispensable variable for achieving full metabolic integration and optimal system functionality. These results indicate that the synergy between HN-AD bacteria and microalgae is crucial for efficient nitrogen and carbon removal, with light intensity and organic carbon supply being key regulatory factors.

In summary, the IBAS achieved synergistic effects between high light intensity (3#) and a sufficient C/N ratio (15). This combination optimized the metabolic coupling of algae and bacteria. Under high-light conditions, microalgae enhanced the O_2_/CO_2_ cycle. At the same time, HN-AD bacteria efficiently degraded NH_4_^+^-N and COD. As a result, the system achieved stable and effective removal of NH_4_^+^-N, TN, and COD. No nitrite accumulation was observed. In contrast, a low C/N ratio (2#) disrupted the carbon nitrogen oxygen exchange between bacteria and algae. This impairment reduced the stability of pollutant removal, even under low light intensity. Beyond confirming the system feasibility, this study highlighted the promise of light-driven bacterial algal biofilm systems. They offer an energy-efficient alternative for wastewater treatment. These systems significantly cut external aeration demands while maintaining robust nitrogen removal and improving system resilience.

It should be noted that a complete nitrogen mass balance distinguishing between assimilatory uptake and dissimilatory denitrification was not performed in this study. Based on the substantial enrichment of HN-AD functional bacteria (e.g., *Acinetobacter*) and the relatively modest biomass yield observed in reactor 3#, dissimilatory denitrification is hypothesized to be the dominant nitrogen removal pathway. Regarding the light intensity applied (335.36 μmol/(m^2^ · s)), direct photosynthetic parameters such as Fv/Fm were not monitored; however, the sustained high pollutant removal performance and stable algal biomass suggest the absence of significant photoinhibition. This intensity falls within the reported optimal range for *Chlorella* and *Halochlorella* (200–400 μmol/(m^2^ · s)) [25,48], whereas photoinhibition is typically reported above 500–600 μmol/(m^2^ · s). Future studies will employ ^15^N isotope labeling and real-time chlorophyll fluorescence monitoring to precisely quantify the nitrogen removal pathways and determine the photoinhibition threshold [49], enabling more refined optimization of the IBAS.

### 2.4. Microbial Community

#### 2.4.1. Sequencing Results and Analysis of α-Diversity Indices and Microbial Evenness

To investigate the effect of IBAS on microbial community structure, samples were collected from reactors 1#, 2#, and 3# after stable operation, which were named S1, S2, and S3 respectively. Additionally, the original sludge sample was named S0. High-throughput sequencing of 16S and 18S rRNA genes was performed on the samples to analyze the changes in bacterial community structure and the evolution of fungal community structure. High-throughput sequencing was performed on 16S and 18S rRNA genes. The results revealed systematic changes in the microbial community structure in response to different C/N ratios and light intensities. These changes highlighted a functionally relevant trade-off between microbial diversity and dominance, which varied with the operating mechanism. As shown in Table 1, the bacterial community of S2 maintained relatively high species richness and diversity, similar to the original sludge (S0), indicating limited selective pressure and a balanced taxonomic profile. In contrast, the diversity of S1 decreased significantly with a marked increase in dominance, which indicated that the carbon-rich and light-exposed conditions exerted strong selectivity on specific HN-AD bacteria. Notably, S3 enriched rare taxa but did not fully restore core diversity, indicating the concurrent occurrence of light-induced diversity and niche specialization. The eukaryotic community showed distinct dynamics. Under high carbon availability, S1 supported the highest microalgal richness. In contrast, S2 severely restricted overall eukaryotic diversity. Despite having low richness, S3 achieved the highest eukaryotic diversity and dominance under high light. This indicated that photosynthetic taxa were stimulated in S3, while non-algal eukaryotes were likely outcompeted. Collectively, these results indicated that light and carbon sources were key regulatory factors for algae bacteria microbial community interactions. These factors formed an interaction network that connected bacteria and microalgae, thereby providing an ecological basis for optimizing bioreactor performance through the adjustment of operating parameters.

#### 2.4.2. Microbial Community Structure Analysis

**Figure 4:**
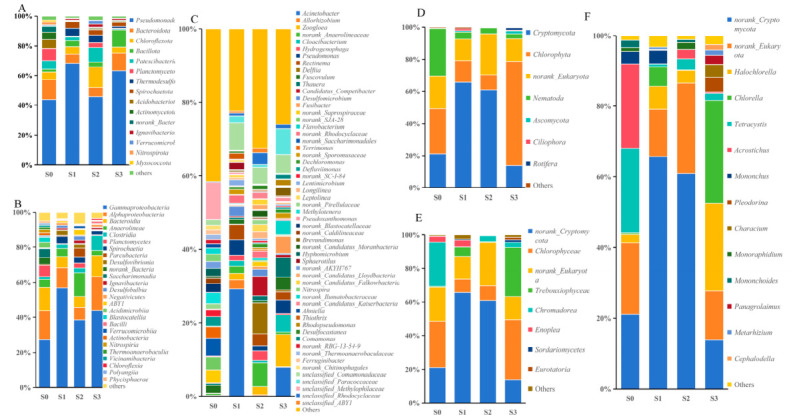
Microbial community structure, bacteria: (**A**) phylum level, (**B**) class level, (**C**) genus level. Eukaryotes: (**D**) phylum level, (**E**) class level, (**F**) genus level.

Operational taxonomic units (OTUs) with a relative abundance >1% were classified as dominant. At the phylum level (Figure 4A), *Pseudomonadota* increased significantly under high C/N ratios. Its abundance rose from 43.8% in S0 to 68.2% in S1 and 63.3% in S3. This increase indicated that *Pseudomonadota* held a strong competitive advantage in high C/N environments, suggesting a key role in HN-AD processes [50]. Conversely, S2 exhibited lower abundance (45.8%) at low C/N ratios, consistent with the hypothesis that carbon source limitation restricts the growth of heterotrophic denitrifying bacteria. A significant enrichment of *Chloroflexota* was observed in S2 (13.8%). A dramatic decline of *Planctomycetes* was observed across all reactors, indicating that the system conditions were unfavorable for these anaerobic ammonium-oxidizing bacteria. Instead, the results suggested that metabolic pathways suited for nutrient-limited conditions may have dominated [51].

At the class level, 26 dominant classes were identified, as shown in Figure 4B. The wastewater treatment system contained numerous common classes, including *Alphaproteobacteria* and *Gammaproteobacteria*, as well as *Clostridia*, *Bacteroidia*, and *Sphingobacteriia*. The *Gammaproteobacteria*, as a diverse group of HN-AD bacteria, accounted for 56.9% in S1 and maintained a high abundance of 43.9% in S3. The relative decline in *Gammaproteobacteria* in S3 suggested that enhanced light exposure may have triggered niche redistribution. *α-Alphaproteobacteria*, the most abundant group in S3 (19.3%), indicated that light exposure may have stimulated functional expression in this phylum.

At the genus level (Figure 4C), *Acinetobacter*, a typical HN-AD bacterium, showed significant proliferation in S1 (from 0.07% in S0 to 29.3%), indicating its pivotal role in denitrification under these conditions. However, its proportion decreased to 7.97% in S3, suggesting that high light exposure may introduce competing microorganisms or alter its ecological niche. The *Zoogloea*, another aerobic denitrifying bacterium, was most abundant in S3 (8.7%), indicating its potential importance in biofilm systems with intense light exposure. The *unclassified_Comamonadaceae* existed stably under all conditions, indicating that it was a core group with continuous denitrification function. The extremely low proportion of *Nitrospira*, a genus of autotrophic bacteria further confirmed that heterotrophic nitrification was predominant in this system [52].

Eukaryotic communities also demonstrated light-dependent characteristics, as shown in Figure 4. In high-light S3, microalgae (*Chlorella* and *Halochlorella*) were highly abundant, which might have facilitated bacterial nitrification/denitrification processes through oxygen supply and organic compound secretion [25,53]. In low-light conditions (S1 and S2), fungi (*Cryptomycota*) dominated, potentially altering metabolic pathways and reducing the efficiency of bacterial algal denitrification synergy.

In conclusion, the carbon-to-nitrogen ratio and light intensity are key environmental factors driving microbial community succession and denitrification function changes. High C/N ratios promote the enrichment of HN-AD microbial communities, while light regulates eukaryotic composition and algal bacterial interaction patterns, further influencing the efficiency and stability of nitrogen transformation pathways. The results showed that the denitrification performance of the immobilized bacterial and algal system can be optimized by regulating the above parameters.

### 2.5. Metabolic Analysis

Metabolomic analysis was performed on the effluent samples from 1#, 2#, and 3#, with a focus on identifying differential metabolites and conducting metabolic pathway enrichment analysis between samples 1 and 2, as well as 1 and 3.

The number of differential metabolites detected between samples with different carbon-to-nitrogen (C/N) ratios was 1111, while that between samples under different light conditions was 682. Differential metabolites were screened according to the HMDB primary classification, and significantly altered metabolites with fold-change >10 were selected for further analysis.

In samples with different C/N ratios, two metabolites were significantly upregulated: *valyl-γ-glutamate* (82.77-fold increase) and *arginyl-glycyl-glutamyl-serine* (18.79-fold increase). *Valyl-γ-glutamate*, due to the stability conferred by its *γ-glutamyl* bond, can serve as a nitrogen storage reservoir for microorganisms. Under nitrogen-limited conditions, its hydrolase releases free glutamate and valine, which can enter the TCA cycle or act as amino donors. This indicates that in high-C/N environments, microorganisms utilize this compound to efficiently store limited environmental nitrogen, while also regulating intracellular osmotic pressure and stabilizing cellular physiological state. This adaptation to a high-carbon, low-nitrogen nutrient environment helps maintain normal metabolism and growth of the strain, representing a key response mechanism of microorganisms to nitrogen limitation.

In samples under different light conditions, ten metabolites were found to be markedly upregulated, primarily including amino acids, carbonyl compounds, *terpene glycosides*, carbohydrates, and hydrolyzable tannins. Among them, *seryl-prolyl-arginyl-p-nitroanilide* showed the highest upregulation (42.09-fold). This compound functions as an osmoregulatory substance in microorganisms. Its metabolic process is closely coupled with the urea cycle of arginine, enabling it to scavenge reactive oxygen species, regulate algal bacterial metabolic pathways, stabilize intracellular enzyme activity and membrane structure, thereby adapting to high-light conditions and ensuring synergistic metabolic functions between algae and bacteria.

**Figure 5:**
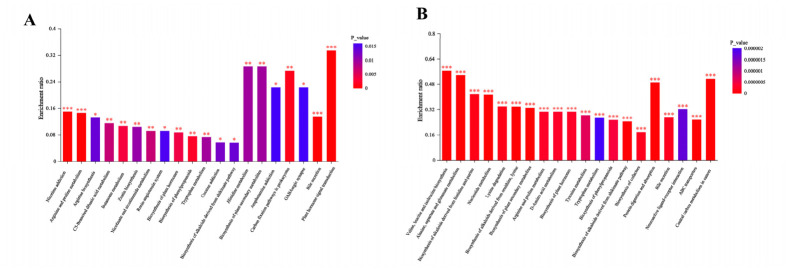
KEGG pathway enrichment characteristics: (**A**) Different light intensities. (**B**) Different C/N. Values with *p*-value or FDR < 0.001 are marked as ***, those with *p*-value or FDR < 0.01 as **, and those with *p*-value or FDR <0.05 as *.

Metabolic pathway enrichment analysis was conducted using the KEGG database. Figure 5 illustrate the enrichment profiles of samples collected under different C/N ratios and light intensities in KEGG pathways. The vertical axis represents the enrichment ratio, defined as the number of enriched metabolites in a given pathway divided by the total number of metabolites annotated to that pathway, reflecting the degree of enrichment of differential metabolites in each pathway. The color gradient indicates the significance of enrichment (*p*-value). The top 20 KEGG pathways enriched with differential metabolites under different C/N ratios included nine pathways related to amino acid metabolism. Among these, the biosynthesis of *valine*, *leucine*, and *isoleucine*; *alanine*, *aspartate*, and *glutamate* metabolism; protein digestion and absorption; and central carbon metabolism exhibited relatively high enrichment ratios.

The top 20 differential metabolic pathways identified under different light conditions included three pathways associated with plant metabolism, three with amino acid metabolism, and two with carbon fixation. Pathways such as *histidine metabolism*, *biosynthesis of plant secondary metabolites*, *plant hormone signal transduction*, and *carbon fixation pathways in prokaryotes* showed higher enrichment ratios.

## 3. Materials and Methods

### 3.1. Test Water and Sludge

The activated sludge used in the experiment was sourced from a municipal wastewater treatment plant in Nanyang City. The relevant parameters of the sludge are as follows: the sludge settling velocity at 30 min (SV) was approximately 30%, the initial mixed liquor suspended solids (MLSS) concentration was about 4322 mg/L, the mixed liquor volatile suspended solids (MLVSS) concentration was about 3457 mg/L, the ratio of MLVSS/MLSS was approximately 0.8, and the sludge volume index at 30 min (SVI) was 64.86.

Synthetic wastewater was prepared using anhydrous sodium acetate (CH_3_COONa) and potassium dihydrogen phosphate (KH_2_PO_4_) as sources of chemical oxygen demand (COD) and Total Phosphorus (TP). Ammonium chloride (NH_4_Cl), potassium nitrate (KNO_3_), and sodium nitrite (NaNO_2_) served as nitrogen sources. The trace element mixture (per liter) comprises: 500 mg of H_3_BO_3_, 400 mg of MnSO_4_ · 4H_2_O, 400 mg of ZnSO_4_ · 7H_2_O, 200 mg of Na_2_MoO_4_ · 4H_2_O, 100 mg of CuSO_4_ · 5H_2_O, 100 mg of CoCl_2_, 100 mg of KI, and 100 mg of NiCl_2_.

### 3.2. Preparation of Immobilized Microbial Ceramic Beads

Ceramic beads with uniform particle size and intact surfaces were selected. After repeated rinsing with deionized water to remove surface impurities, they were sterilized at 121 °C in an autoclave for 20 min, cooled to room temperature, and dried. Subsequently, appropriate amounts of the ceramic beads were added to the cultured *Pseudomonas alcaliphila* bacterial suspension and *Paracoccus versutus* bacterial suspension, respectively. The mixtures were then placed in an incubator shaker at 38 °C with a shaking speed of 150 rpm for 24 h to allow microbial adhesion and biofilm formation on the ceramic beads via natural adsorption and proliferation.

### 3.3. Experiment Setup

#### 3.3.1. Algae Growth Investigation

Four comparative experiments were conducted in 250 mL shake flasks to observe algal growth under different light and container sealing conditions. Before the experiment, equal amounts of ceramic beads loaded with bacteria were added to each shake flask. The experimental design included four groups: P1 (sealed under natural light), P2 (sealed in dark), P3 (open under natural light), and P4 (open in dark). Sealed flasks were fitted with sterile butyl rubber stoppers and wrapped with Parafilm M^®^ (Beijing Qiwei Yicheng Technology Co., Ltd., Beijing, China) to ensure gas-tightness, while open flasks were covered with sterile breathable sealing film to allow gas exchange while preventing contamination. Two replicates were set up for each condition. Algal growth was monitored over a 30-day period divided into three successive phases: Phase I (1–10 days, growth initiation), Phase II (11–20 days, accelerated proliferation), and Phase III (21–30 days, stable growth period). For the sealed groups (P1 and P2), the 250 mL shake flasks were fitted with sterile butyl rubber stoppers and further sealed with Parafilm M^®^ wrapping around the cap flask junction. Gas-tightness was verified before the experiment by submerging the sealed flasks in water and confirming the absence of air bubble leakage. For the open groups (P3 and P4), flasks were covered with sterile breathable sealing film (AeraSeal^®^, Shandong Barok Biotechnology Co., Ltd., Jinan, China) to allow gas exchange while preventing microbial contamination. Two replicates were set up for each condition, and the changes in the bottle were observed every ten days.

#### 3.3.2. IBAS Investigation on Pollutant Removal Efficiency for Nitrogen Sources with Varying Concentrations and Forms

A series of batch experiments were conducted in 250 mL Erlenmeyer flasks to evaluate denitrification performance under different nitrogen sources and concentrations. The experimental design was divided into three groups based on nitrogen sources: sample 1 ammonium nitrogen (NH_4_^+^-N), sample 2 nitrate nitrogen (NO_3_^−^-N), and sample 3 nitrite nitrogen (NO_2_^−^-N). Each group was prepared with three initial concentration gradients: 50, 70, and 100 mg/L. All flasks were filled with synthetic wastewater (see Table 2 for detailed composition), with an initial chlorophyll-a concentration of approximately 5 mg/L, to simulate algae-rich water. In each flask, a certain amount of activated sludge (obtained from the local municipal sewage treatment plant) was inoculated as a microbial inoculum. Furthermore, ceramic beads loaded with HN-AD bacteria were used as bio-enhancers to enhance the denitrification process.

All experiments were conducted in triplicate (*n* = 3) to ensure statistical reliability.

#### 3.3.3. Investigation on the Influence of Various Factors on IBAS Denitrification

Based on the optimal nitrogen source and dosage concentration determined in shake flask experiments, an SBR reactor was used to study the effects of different influencing factors on the decontamination effect. The other conditions of each reactor are as follows: 1# (C/N = 15:1, light intensity 134.3 μmol/(m^2^ · s)), 2# (C/N = 8:1, 134.3 μmol/(m^2^ · s)), 3# (C/N = 15:1, light intensity 335.36 μmol/(m^2^ · s)). The system employed full light environment isolation throughout the entire process, with water temperature maintained at 25 ± 2 °C. At the start of the experiment, 1 L of sludge and 90 g of immobilized functional microbial ceramic beads were added to each reactor. Water samples were collected from the outlet every two days. Parameters including COD, NH_4_^+^-N, NO_2_^−^-N, NO_3_^−^-N, and TN were measured.

### 3.4. Indicator Detection and Statistical Analysis

Water quality parameters (NH_4_^+^-N, NO_2_^−^-N, NO_3_^−^-N, and TN) and sludge physicochemical parameters (MLSS, MLVSS, SV30, and SVI30) were all measured using the standard method [54]. The COD rapid measurement system (HACH, Loveland City, CO, USA) was used to measure COD [54]. The pH measurement was performed using a pH meter (HC3800 sc, Loveland City, CO, USA). DO was measured using a dissolved oxygen meter (HQ40D, HACH Water Quality Analytical Instrument Co., Ltd., Loveland City, CO, USA).

All statistical analyses were performed using SPSS 15.0. A paired t-test was employed to evaluate the denitrification and decarbonization efficiency of immobilized particles across samples based on *p*-values, and to determine whether significant differences existed among different sludge systems and operating conditions. A *p*-value < 0.05 was considered significant.

### 3.5. High-Throughput Sequencing

On the final day of each experimental cycle, appropriate quantities of sludge were collected as test samples. The samples obtained from tanks 1#,2#, and 3# were labeled as S1, S2, and S3 respectively. Immediately after sampling, the samples were frozen and sent to Shengong Bioengineering (Shanghai, China) Co., Ltd. for Illumina MiSeq high-throughput sequencing.

Using the genomic DNA of the aforementioned samples as templates, PCR amplification was performed for the V3–V4 region of bacterial 16S rRNA using B341F and B785R primers, and for the 18SV4 region of fungal 18S rRNA using 18SV4F and 18SV4R primers. The high-throughput sequencing data underwent quality control using Cutadapt (v1.9.1), Prinseq (v0.20.4), and Flash (1.2.3) software. The validated sequences were then subjected to operational taxonomic units (OTUs) analysis at 97% similarity threshold.

The community composition of each sample at different taxonomic levels was statistically analyzed using Mothur (1.30.1) software and the Silva database. Additionally, the coverage rate and microbial alpha diversity indices (including the Simpson, Shannon, ACE, and Chao1 indices) were calculated for each sample.

### 3.6. Metabolite Data Analysis

Metabolomics-related analyses were conducted by Majorbio (Shanghai, China).

The metabolites were identified by searching database, and the main databases were the HMDB (http://www.hmdb.ca/ (accessed on 15 January 2026)), Metlin (https://metlin.scripps.edu/ (accessed on 15 January 2026)) and Majorbio Database. The metabolites with VIP > 1, *p* < 0.05 were determined as significantly different metabolites based on the Variable importance in the projection (VIP) obtained by the OPLS-DA model and the *p*-value generated by the *t*-test. Differential metabolites among two groups were mapped into their biochemical pathways through metabolic enrichment and pathway analysis based on KEGG database (http://www.genome.jp/kegg/ (accessed on 15 January 2026)). Enrichment analysis was used to analyze a group of metabolites in a function node whether appears or not. The principle was that the annotation analysis of a single metabolite develops into an annotation analysis of a group of metabolites. Python 3.12 packages “scipy.stats 1.16.0” (https://docs.scipy.org/doc/scipy/ (accessed on 15 January 2026)) were used to perform enrichment analysis to identify the most relevant biological pathways for the experimental treatments.

## 4. Conclusions

This study constructed an immobilized bacteria algae symbiotic system (IBAS) to investigate nitrogen removal under varying nitrogen sources, C/N ratios, and light intensities. The system achieved exceptional removal efficiencies (≥95%) for both nitrite and nitrate nitrogen. Under high C/N ratio (15:1) and high light intensity (335.36 μmol/(m^2^ · s)), consistent removal rates exceeding 90% for NH_4_^+^-N, TN, and COD were maintained without NO_2_^−^-N accumulation. Microbial community analysis revealed that a high C/N ratio enriched HN-AD bacteria (e.g., within the phylum *Pseudomonadota*), while high light intensity increased microalgal biomass (*Chlorella*), enhancing algae bacteria oxygen carbon cycling. The results demonstrated that regulating C/N ratio and light intensity can effectively optimize the microbial community and system function, offering a novel strategy for low-carbon wastewater treatment.

## Figures and Tables

**Table 1:** Sequencing results and diversity indices of species diversity and richness index analysis.

Operating Conditions	Sample	Number	OTUs	Ace	Chao	Shannon	Simpson	Coverage
16S	S0	171,799	1393	1428.9496	1418.6667	5.395251	0.016178	0.999552
	S1	73,676	1284	1468.6973	1391.9061	4.21301	0.08713	0.996675
	S2	78,298	1434	1554.499	1508.025	5.340763	0.014828	0.997586
	S3	70,733	1526	1436.7229	1364.0037	4.744095	0.021646	0.996579
18S	S0	103,312	96	100.4506	98	2.0709	0.1756	0.9999
	S1	117,853	122	123.5638	123.4285	1.7265	0.382	0.9999
	S2	129,703	84	90.0024	87.75	1.4929	0.3738	0.9999
	S3	60,750	85	87.1535	86.6666	2.2653	0.5099	0.9999

**Table 2:** Different components simulate the water quality composition of sewage.

Group	NH_4_^+^-N (mg/L)	NO_3_^−^-N (mg/L)	NO_2_^−^-N (mg/L)	TN (mg/L)	COD (mg/L)	TP (mg/L)
Sample 1	50 ± 2	na	na	50 ± 2	750 ± 5	10 ± 0.1
	70 ± 3	na	na	70 ± 3	1050 ± 8	14 ± 0.2
	100 ± 3	na	na	100 ± 3	1500 ± 10	20 ± 0.3
Sample 2	na	50 ± 0.6	na	50 ± 0.6	750 ± 5	10 ± 0.1
	na	70 ± 0.8	na	70 ± 0.8	1050 ± 8	14 ± 0.2
	na	100 ± 1.1	na	100 ± 1.1	1500 ± 10	20 ± 0.3
Sample 3	na	na	50 ± 0.1	50 ± 0.1	750 ± 5	10 ± 0.1
	na	na	70 ± 0.2	70 ± 0.2	1050 ± 8	14 ± 0.2
	na	na	100 ± 0.2	100 ± 0.2	1500 ± 10	20 ± 0.3

na: not applicable; NH_4_^+^-N: Ammonium-nitrogen; NO_3_^−^-N: Nitrate-nitrogen; NO_2_^−^-N: Nitrite-nitrogen; TP: Total phosphorus. Values are presented as the mean ± standard deviation obtained from two independent experiments.

## Data Availability

Data will be made available on request.

## Abbreviations

The following abbreviations are used in this manuscript:

IBAS	Immobilized Bacteria Algae Symbiotic System
HN-AD	Heterotrophic Nitrification Aerobic Denitrification
C/N	Carbon to Nitrogen ratio
COD	Chemical Oxygen Demand
TN	Total Nitrogen
TP	Total Phosphorus
DO	Dissolved Oxygen
FA	Free Ammonia
AMO	Ammonia Monooxygenase
HAO	Hydroxylamine Oxidase
NAP	Periplasmic Nitrate Reductase
NIR	Nitrite Reductase
OTU	Operational Taxonomic Unit
MLSS	Mixed Liquor Suspended Solids
MLVSS	Mixed Liquor Volatile Suspended Solids
SV	Sludge Settling Velocity
SVI	Sludge Volume Index
